# Discovery of urinary biomarkers to discriminate between exogenous and semi-endogenous thiouracil in cattle: A parallel-like randomized design

**DOI:** 10.1371/journal.pone.0195351

**Published:** 2018-04-12

**Authors:** Lieven Van Meulebroek, Jella Wauters, Beata Pomian, Julie Vanden Bussche, Philippe Delahaut, Eric Fichant, Lynn Vanhaecke

**Affiliations:** 1 Laboratory of Chemical Analysis, Department of Veterinary Public Health and Food Safety, Faculty of Veterinary Medicine, Ghent University, Salisburylaan, Merelbeke, Belgium; 2 Health Department, CER Groupe, Rue Point du Jour, Marloie, Belgium; University of Illinois, UNITED STATES

## Abstract

In the European Union, the use of thyreostats for animal fattening purposes has been banned and monitoring plans have been established to detect potential abuse. However, this is not always straightforward as thyreostats such as thiouracil may also have a semi-endogenous origin. Therefore, this study aimed at defining urinary metabolites, which may aid in defining the origin of detected thiouracil. Hereto, a parallel-like randomized *in vivo* study was conducted in which calves (n = 8) and cows (n = 8) were subjected to either a control treatment, rapeseed-enriched diet to induce semi-endogenous formation, or thiouracil treatment. Urine samples (n = 330) were assessed through metabolic fingerprinting, employing liquid-chromatography and Q-Exactive^TM^ Orbitrap mass spectrometry. Urinary fingerprints comprised up to 40,000 features whereby multivariate discriminant analysis was able to point out significant metabolome differences between treatments (Q^2^(Y) ≥ 0.873). Using the validated models, a total of twelve metabolites (including thiouracil) were assigned marker potential. Combining these markers into age-dependent biomarker panels rendered a tool by which sample classification could be improved in comparison with thiouracil-based thresholds, and this during on-going thiouracil treatment (specificities ≥ 95.2% and sensitivities ≥ 85.7%), post-treatment (sensitivities ≥ 80% for ≥ 24 h after last administration), and simulated low-dose thiouracil treatment (exogenous thiouracil below 30 ng μL^-1^). Moreover, the metabolic relevance of revealed markers was supported by the suggested identities, for which a structural link with thiouracil could be determined in most cases. The proposed biomarker panels may contribute to a more justified decision-making in monitoring thiouracil abuse.

## Introduction

In livestock, the administration of thyreostats has been associated with a significant weight gain, which is primarily due to an increased water retention by the edible tissues and filling of the gastrointestinal tract. As such, these growth-promoting agents exert a negative effect on the quality of meat [1]. Moreover, xenobiotic thyreostats have been assigned teratogenic and carcinogenic properties (group 2b, IARC) [2], implicating that any residues in consumable matrices may connote a possible risk for human health. Therefore, the use of thyreostats for animal fattening purposes has been banned in the European Union since 1981 [3]. This ban implied a strict zero-tolerance policy with respect to the use of thyreostatic drugs and the presence of their residues in derived animal matrices (i.e. milk, urine, muscle and organ tissue). This resulted in a constant monitoring by the European Union member states in order to discourage thyreostat abuse, being at risk of severe penalties. In this context, it may be noted that administration of thyreostats has become rather uncommon because of the fast elimination kinetics and inefficient growth-promoting actions [4,5]. As such, the use of growth promotors at very low doses in combination with other drugs, aiming at additional or synergistic effects, has become more popular in the last decade [6,7].

In recent years, a semi-endogenous origin of the thyreostat thiouracil (TU) has been reported in urine of livestock upon ingestion of glucosinolate-rich crops, belonging to the Brassicaceae family [8,9,10]. The discovery of this semi-endogenous origin was a startling finding as the rigid link between detection of urinary TU and its prior use was no longer binding. Moreover, this finding was able to explain the presence of low TU levels in urine from livestock, for which there were no direct indications of illegal administration [11]. Although the exact mechanisms of endogenous TU formation and the (dietary) triggers have not been fully uncovered yet [12], it may be clear that the existence of a semi-endogenous origin strongly impedes the decision-making process concerning a potential illegal use of TU upon its detection. In this regard, the community of the European Reference Laboratories has suggested urinary TU concentration levels for which a semi-endogenous origin may be presumed, i.e. below 10 μg L^-1^ [13]. As such, a recommended concentration of 10 μg L^-1^ or 10 μg kg^-1^ was introduced, which was accompanied with specific analytical requirements for the measurement of certain thyreostats, i.e. TU in urine and thyroid tissue [13]. Unfortunately, a systematic occurrence of urine samples, exceeding this recommended TU concentration, has been observed in recent years [14,15,16]. Therefore, national and international surveys have been conducted to set a new threshold value that is more accurate to differentiate between exogenous and semi-endogenous TU [5]. More specifically, a recommended TU concentration of 30 μg L^-1^ was suggested, which is discussed in the latest reflection paper of the European Reference Laboratories [17]. However, even with this updated threshold, incorrect decision-making with false compliants or non-compliants is still possible. This could for example be the case for young male bovines (6 to 12 months) for which the international 99^th^ percentile TU threshold value was determined to be 35.9 μg L^-1^; thus exceeding the newly recommended TU concentration [5]. As a consequence, an alternative strategy to discriminate between exogenous and semi-endogenous thiouracil is highly demanded. In this regard, untargeted profiling techniques may enclose significant value as these could reveal marker molecules that are descriptive for the origin of detected TU. Such a biomarker approach was already proven effective to unequivocally point out steroid treated animals, i.e. livestock being administered with prednisolone [18], 4-androstenedione [19], 17β-nandrolone laureate esters and 17β-estradiol 3-benzoate [20], or nadrolone [21]. Hereby, adequate analytical techniques, including nuclear magnetic resonance or full-scan mass spectrometry (time of flight, Orbitrap or Fourier transform ion cyclotron resonance), are most suited to perform global metabolome analyses [22].

In this study, it was aimed to reveal a urinary biomarker or marker signature, which is indicative for exogenous TU administration in bovines and thus able to uncover the true origin of detected TU. To this extent, a comprehensive *in vivo* study was carried out in which calves and adult cows were subjected to various treatments with the intention to obtain urine samples with either no TU (control diet), semi-endogenous TU (rapeseed-enriched diet) or exogenous TU (control diet and oral TU administration). To map the urinary metabolomes, a method for metabolic fingerprinting (using ultra-high performance liquid chromatography (UHPLC) and high-resolution hybrid quadrupole Orbitrap mass spectrometry (HRMS/MS)) was developed.

## Materials and methods

### Experimental set-up of the *in vivo* trial

**Fig 1:**
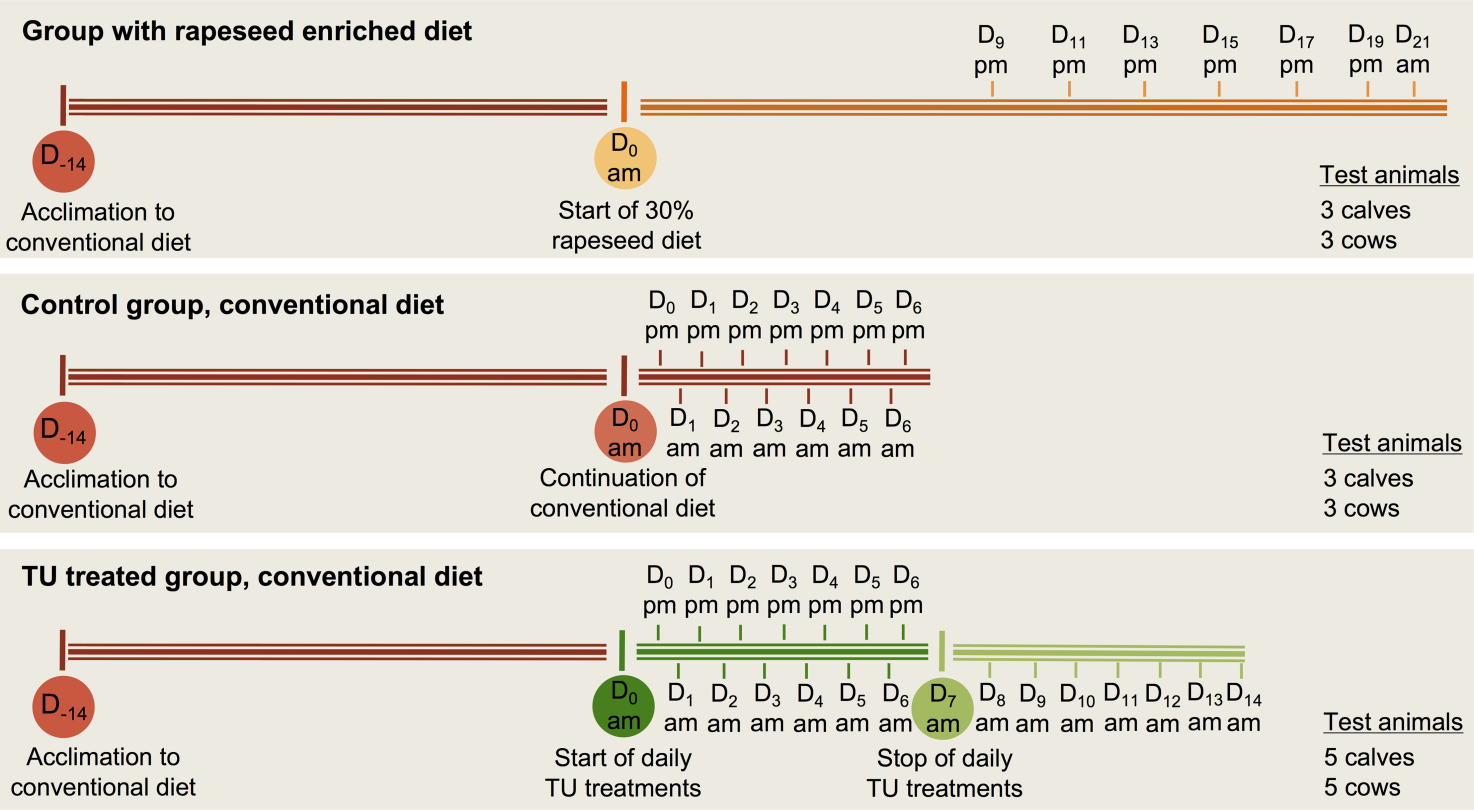
Schematic representation of the *in vivo* trial. The small ticks in the scheme represent urine sampling moments, being relatively expressed towards treatment starting points (i.e. days (D), morning (am), afternoon (pm)).

The *in vivo* trial concerned a parallel-like design with various treatment groups, as depicted in Fig 1. Each of the treatments was preceded by a 2-week acclimation phase during which all test animals were fed a commercial diet of concentrate (27% crude protein content) with *ad libitum* access to water and hay. Whereas this dietary regimen was maintained for the control and TU treated group, one fraction of the test animals received a diet in which 30% of the concentrate was replaced by rapeseed cake (37% crude protein content), with the aim of inducing the semi-endogenous formation of TU. With respect to the TU treated group, animals were administered a daily dose of 0.2 g TU per 100 kg body weight (TU analytical powder, Sigma Aldrich, St. Louis, MO, USA) by means of a filled capsule. This was realized using a capsule launcher and flushing the capsule with water through the mouth and esophagus into the stomach. Following the 1-week TU treatment, a short washout period was appended to assess the excretion profiles of potential markers in urine.

Test animals were housed under controlled experimental conditions at the animal facilities of Centre d’Economie Rurale (CER, Marloie, Belgium), whereby animals were kept in separate half-covered pens. It was hereby opted to include both cows and calves since an age-dependent metabolic response towards TU treatment could be expected. All calves (female, 101.6 ± 14.7 kg, 3.3 ± 0.3 months) were of a mixed breed, whereas cows (female, 587.1 ± 98.9 kg, 4.0 ± 1.5 years) were either of a milk or meat breed. The number of animals within each treatment group is presented in Fig 1. This *in vivo* study was approved by CER’s Ethical Committee (CE/Santé/ET004).

### Chemicals and reagents

The analytical standards 2-thiouracil (TU), 6-dimethyl-thiouracil, 6-ethyl-thiouracil, 6-methyl-2-thiouracil, 6-propyl-2-thiouracil, and 6-phenyl-thiouracil were purchased from Sigma-Aldrich (St. Louis, MO, USA), whereas the deuterated internal standard 6-propyl-2-thiouracil-d_5_ (PTU-d_5_) was from Toronto Research Chemicals (Toronto, Canada). Stock (1 mg mL^-1^) and working solutions (1 and 0.1 ng μL^-1^) were prepared in methanol and stored in dark glass bottles at -20°C.

Reagents were of analytical grade when used for extraction purposes and of LC-MS grade for UHPLC-MS applications. They were respectively purchased from VWR International (Merck, Darmstadt, Germany) and Fisher Scientific (Loughborough, UK). Ethylenediaminetetraacetic acid (EDTA) was from VWR International (Merck, Darmstadt, Germany) and hydrogen chloride from Sigma-Aldrich (St. Louis, MO, USA). Phosphate buffer was adjusted to a pH of 7 and saturated with 1% of DL-dithiothreitol (DTT) (Sigma-Aldrich, St. Louis, MO, USA). Ultrapure water (0.055 μS cm^-1^) was obtained by means of a purified-water system (Sartorius AG, Göttingen, Germany).

### Sample collection, preservation and extraction

Urine samples were collected during feeding upon spontaneous micturition, while carefully avoiding fecal contamination. Urine was divided into several 5-mL aliquots and treated with EDTA (final concentration of 0.1 M) and 0.1 M hydrogen chloride (final pH of 1) to inhibit thyreostat degradation during urine storage [23]. Urine samples were stored at -20°C for about one and a half year until metabolomics analyses were performed and thereby thawed at 4°C prior to extraction. For this relatively long storage period, changes in the metabolic profile can be assumed as reported by Laparre et al. (2017) [24]. However, these changes were presumed to be independent of the type of urine sample, corresponding to the various treatments. Yet, degradation of certain compounds to levels below their limits of detection may have reduced the initial pool of candidate markers, which also implies that any markers that were retained in this study are likely to be stable or have low limits of detection.

For development of the extraction protocol, a pool of urine from nine bovines was considered. These bovines were not treated with TU nor received a rapeseed-enriched diet and were not part of the experiment as described in Fig 1. Various procedures (S1 Text) were assessed for their ability to efficiently extract the urinary metabolome, whereby the total number of detectable metabolite features served as the main indicator of metabolome coverage. Additionally, a targeted focus on thyreostats was included to respond to the specific research question. The final extraction protocol was based on the methodology of Vanden Bussche *et al*. (2010) [25] with some minor modifications. In brief, 3 mL of urine was enriched with 50 ng PTU-d_5_ internal standard and supplemented with 1 mL phosphate buffer, containing 1% of the reducing agent DDT. Hereby, denaturing conditions (65°C, 30 min) were imposed to impede protein-thyreostat interactions and avoid protein-based interference. Next, a two-fold liquid-liquid extraction with 5 mL ethyl acetate was performed, after which the pooled supernatants were evaporated to dryness at 60°C under a gentle stream of nitrogen. The remaining residue was dissolved in 200 μL ultrapure water (0.1% formic acid) and methanol (0.1% formic acid) (90/10, v/v). The injection volume was 10 μL. This protocol can be consulted on protocols.io (dx.doi.org/10.17504).

### Liquid chromatography and mass spectrometry

Optimization of the UHPLC method was based on a selection of relevant thyreostats, described in section “Chemicals and reagents”, whereby the achieved chromatographic resolution (R_s_), peak shape (A_s_), and peak intensity were the main evaluated performance criteria. In first instance, the selectivity of various C18 columns was assessed, i.e. by considering the Hypersil Gold (1.8 μm, 2.1 x 100 mm) (Thermo Fisher, San Jose, USA), Kinetex (1.7 μm, 2.1 x 150 mm) (Phenomenex, Torrence, CA, USA), and Acquity HSS T3 (1.8 μm, 2.1 x 100 mm) (Waters, Manchester, UK) column. In addition, various organic solvents (acetonitrile and methanol), modifiers (0.1% formic acid and 6.5 mM ammonium bicarbonate), gradient programs, flow rates and column temperatures were tested. The UHPLC system consisted of a Dionex Ultimate 3000 XRS pump, coupled to a Dionex Ultimate 3000 RS column compartment and autosampler (Dionex, Amsterdam, The Netherlands). The final methodology achieved chromatographic separation on an Acquity HSS T3 column (1.8 μm, 2.1 x 100 mm) (Waters, Zellik, Belgium), whereby a gradient program using 0.1% formic acid in water (solvent A) and 0.1% formic acid in methanol (solvent B) was applied. Following proportions of solvent A were used: 0–1 min at 90%, 1–3 min from 90 to 79%, 3–5 min from 79 to 20%, 5–9 min from 20 to 0%, 9–12 min at 0%, 12–12.1 from 0 to 90%, followed by 3 min of re-equilibration. A constant flow rate of 300 μL min^-1^ and a column oven temperature of 25°C were set.

Mass spectrometric analysis was carried out using a high-resolution hybrid quadrupole Q-Exactive^TM^ Orbitrap mass spectrometer (Thermo Fisher Scientific, San Jose, USA), which was equipped with a heated electrospray ionization source (HESI-II), operating in polarity switching mode. Instrumental settings for full-scan MS events were optimized based on the thyreostats’ peak areas and signal-to-noise ratios and involved a sheath gas flow rate of 2 arbitrary units (au), an auxiliary gas flow rate of 10 au, a sweep gas flow rate of 2 au, a capillary temperature of 250°C, a heater temperature of 275°C, a spray voltage of (-)3 kV, and an S-lens RF level of 50 au. Mass resolution and automatic gain control (AGC) were determined by analysis of urine extracts, spiked with thyreostats to reach final concentrations of 0.05 ng μL^-1^. Final settings were a mass resolution of 70,000 full width at half maximum (FWHM) and an AGC target of 3 e^6^ ions. The *m/z* scan range was set from 100 to 800 Da. In addition, separate MS/MS fragmentation experiments were performed for identification of revealed markers. These experiments applied parallel reaction monitoring (PRM) with usage of an inclusion list and an AGC target of 1 e^5^ ions, an *m/z* isolation window of 2.0 Da, a mass resolution of 17,500 FWHM, and a collision energy of 20, 35, and 60 eV. Instrumental control and data processing were carried out with Chromeleon Express and XCalibur 3.0 software (Thermo Fisher Scientific, San Jose, USA).

Urine samples that were considered for metabolic fingerprinting have previously (after about a half year of storage) also been analyzed in a targeted fashion using a triple quadrupole instrument, whereby TU concentrations were determined using matrix-matching calibration curves [16]. TU concentrations, as discussed in this manuscript, were thus obtained by the cited targeted approach.

### Metabolic fingerprinting of urine samples

For discovery of candidate biomarkers, the urinary fingerprints of the control group were compared with those of the TU treated group (excl. washout). To this end, urine samples from both treatment groups were extracted and analyzed in a partly random order, keeping the batches of the cows and calves separately. With respect to the randomization, it was opted to alternate between samples from TU treatment and the control group, however, thereby at the same time achieving data acquisition animal per animal (samples from an animal were, however, fully randomized). During the discovery phase, data were interpreted in a truly untargeted fashion, meaning that the complete metabolic fingerprint was taken into consideration to seek for candidate markers. Following this, the classification performance of the selected candidate markers was further evaluated by considering the samples from the rapeseed-enriched diet and washout group. These samples were analyzed in separate batches according to cow or calf, and rapeseed-enriched diet or washout. Hereby, samples from a particular animal were run successively. The acquired full-scan data was processed in a targeted fashion (XCalibur 3.0 software), meaning that only the previously selected candidate markers were considered and semi-quantified. During mass spectrometric analysis of urine extracts, quality control measures were taken by considering external and internal quality control (QC) samples. QC samples were prepared starting from pooled urine (equal volume contributions from at least 60 samples), which was extracted according to the standard protocol. External QC samples were used for instrument stabilization, whereas internal QC samples were included to monitor and correct for instrumental drift.

Acquired full-scan MS data of study samples were imported into the Sieve^TM^ 2.2 software package (Thermo Fisher Scientific, San Jose, USA) to compose metabolic fingerprints. Metabolite features from total ion current chromatograms were extracted, applying peak alignment and integration. As primary parameters, a frame *m/z* width of 6 ppm, a frame time width of 0.75 min, and an intensity threshold of 1,000,000 were set. Each of the detected features was characterized through its retention time (*t*_R_) and *m/z*-value.

### Statistical analysis and data interpretation

For multivariate data analysis, SIMCA 14.1 (Umetrics, Malmö, Sweden) was used, whereby the QC-normalized data matrices served as input data. Data were log-transformed and pareto-scaled to induce normality and standardize the peak intensity ranges, respectively. In first instance, unsupervised segregation was checked by principal component analysis (PCA), allowing to evaluate clustering of QC samples and identify potential outliers. Subsequently, orthogonal partial least squares discriminant analysis (OPLS-DA) was performed to model variation and establish separation between investigated treatment groups [26]. Validity of OPLS-DA models was verified by cross-validation ANOVA (p-value < 0.05), permutation testing (n = 100), and three model characteristics (R^2^(X), R^2^(Y), and Q^2^(Y), calculated by 7-fold internal cross validation) [27]. With respect to the latter, a Q^2^(Y) > 0.5 indicated good model predictability [28]. Validated OPLS-DA models were used to select differentiating metabolites, thereby considering various mathematical descriptors. In first instance, the S-plot was used, considering the contribution of an ion towards class separation (i.e. covariance p) and the reliability of this contribution (i.e. correlation corr(p)). A fixed setting of |p| ≥ 0.05 and varying cut-off value for corr(p) were adopted to establish a first selection or relevant ions [29]. This strategy was supported by the Variable Importance in Projection scores (≥ 1) and Jack-knifed confidence intervals (not across zero) [30,31].

Univariate statistical analyses that were performed within this research used SPSS^®^ Statistics 23 (IBM, USA), assessing the correlation between variables (i.e. Kendall’s τ whereby a value of 0.50 was taken as a minimum to conclude significant correlation) or the equality of population means (i.e. Mann-Whitney nonparametric test, p-value ≤ 0.05 to conclude significant differences).

To integrate the various revealed candidate biomarkers into a single biomarker panel that could be easily used for sample classification, a mathematical descriptor was calculated. Hereby, the strategy and its inherent equations, as proposed by Dervilly-Pinel *et al*. (2015) [32], was applied.

## Results and discussion

### Analytical methodology for polar metabolite screening

The method of Vanden Bussche *et al*. (2010) [25] proved itself as one of the better with respect to metabolome coverage. Indeed, using the urine pool, 34,990 positively and 5,879 negatively charged features were detected, being slightly outperformed (+10.1%) by the best-ranked method. However, the methods that were better ranked in terms of metabolome coverage were not suitable to detect thyreostats. Therefore, the method of Vanden Bussche *et al*. (2010) [25] served as the starting point, whereby further optimization efforts were directed towards an improved metabolome coverage. This was mainly achieved by adjusting the urine starting volume from 1 to 3 mL. As such, the number of detectable features was increased by 7.3%. Based on the dilution series of several thyreostats, potential instrument saturation and lack of linearity were not observed (R^2^ > 0.99).

With respect to the UHPLC method, the Acquity HSS T3 (1.8 μm, 2.1 x 100 mm) stationary phase proved most appropriate to achieve proper separation (R_s_ ≥ 2.07) and symmetric peak shape (A_s_ between 0.8 and 1.2) for all target thyreostats. Furthermore, based on the sensitivity, peak shape, and chromatographic resolution, a definite selection of the organic solvent, modifier, gradient program, flow rate and column temperature were made, with the final settings being presented in the section “Sample collection, preservation and extraction”.

Optimal HESI working conditions, described in section “Liquid chromatography and mass spectrometry”, were determined based on the thyreostats’ peak areas and signal-to-noise ratios. The optimal AGC setting was 3 e^6^ ions, which allowed assuming maximal spectral stability from scan to scan. In addition, based on the improved signal-to-noise ratios and the verified low mass deviations (< 1.65 ppm), significant effects due to space charging were excluded. Mass resolution was optimized by pursuing maximum mass accuracy while retaining a sufficient number of data points across the chromatographic peak. A resolution of 70,000 FWHM was found optimal, being associated with high signal-to-noise ratios and peak areas, while at least 18 data points per peak were acquired.

### Discovery of candidate biomarkers

Urinary fingerprints were established for cows and calves, thereby distinguishing between positively and negatively charged ions. Fingerprints included 39,961 ions for adult cows and 29,128 ions for calves, with the majority of ions obtained in positive ionization mode (i.e. 63.1% for cows and 69.8% for calves). Associated data matrices of peak intensities were normalized based on the internal QC samples.

**Fig 2:**
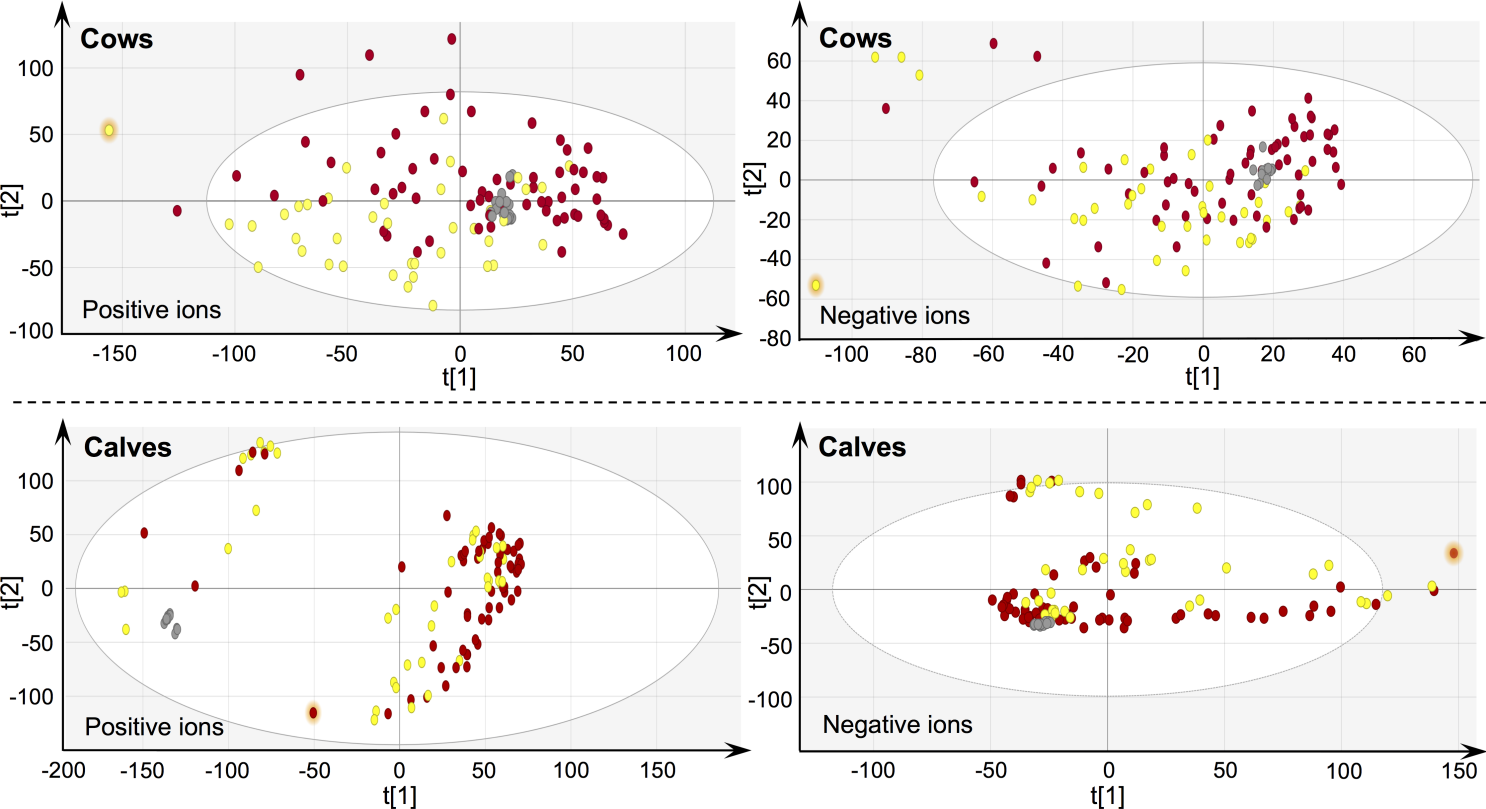
PCA-X score plots. Score plots for cow and calf urine samples that were derived from the TU treated group (n = 65) (red) and untreated control group (n = 39) (yellow), thereby separating between negative and positive ions. Internal quality control samples are colored grey, outliers are highlighted with an orange glow.

PCA-X modelling was performed to evaluate the instrument’s stability during analysis, reveal the natural patterning of samples, and detect potential outliers. PCA-X score plots (Fig 2) pointed at acceptable instrument stability, sample separation in line with treatments, and the presence of some deviating samples. A sample was effectively removed when its deviating behavior was confirmed by the Hotelling’s T^2^ test (99% critical level). It may be noted that samples are more scattered in the case of calves, which may reflect metabolic changes that are associated with growth and maturation [33].

**Fig 3:**
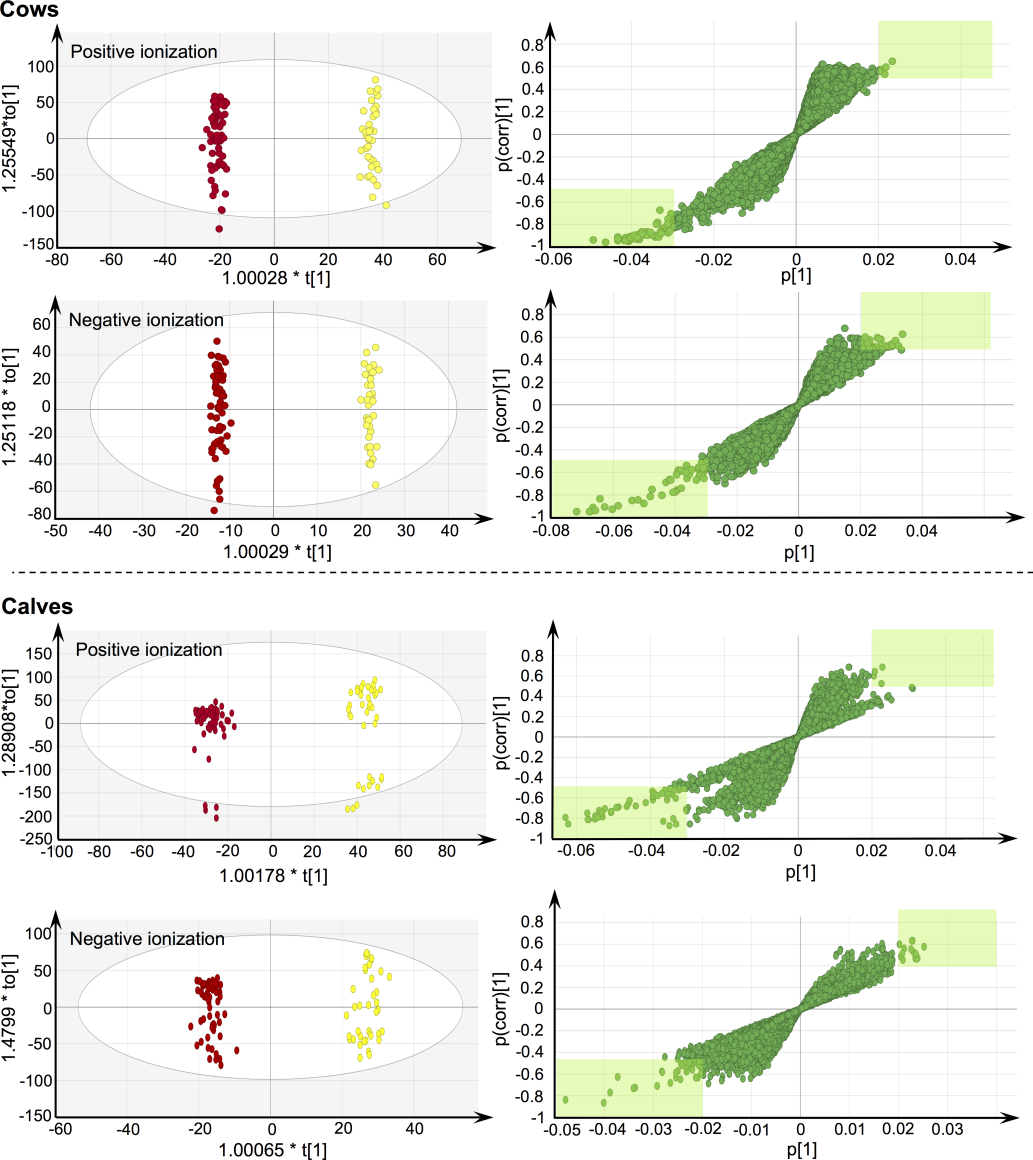
OPLS-DA score plots and associated S-plots. Generated plots visualize the separation between the TU treated group (red) and control group (yellow). With respect to the S-plots, green-colored areas (|p| ≥ 0.05 and corr(p) ≥ +0.02 or ≤ -0.035 or -0.02) indicate ions that were assigned discriminating potential.

Subsequent to PCA-X, metabolic fingerprints were subjected to OPLS-DA modelling to differentiate between the TU treated and control groups. In this context, information about class membership was used. Valid models were generated for calves and adult cows, and this for both ionization modes (Table 1 and Fig 3).

**Table 1:** Specifications of constructed OPLS-DA models and output of model validation, considering control and TU treated animals.

model specifications	number of model components (to + tp)^a^	model characteristics^b^	cross-validated ANOVA (p-value)^c^	permutation testing^d^
cows– positive ions	1 + 5	R^2^(X) = 0.502 R^2^(Y) = 0.996 Q^2^(Y) = 0.883	3.69 e^-33^	Good
cows– negative ions	1 + 8	R^2^(X) = 0.544 R^2^(Y) = 0.997 Q^2^(Y) = 0.891	1.90 e^-32^	Good
calves– positive ions	1 + 5	R^2^(X) = 0.513 R^2^(Y) = 0.985 Q^2^(Y) = 0.873	7.87 e^-33^	Good
calves– negative ions	1 + 7	R^2^(X) = 0.646 R^2^(Y) = 0.988 Q^2^(Y) = 0.894	1.94 e^-34^	Good

^a^ with to the orthogonal and tp the predictive component.

^b^ with R^2^(X) the variation in X that is explained by the model, R^2^(Y) the variation in Y that is explained by the model, and Q^2^(Y) the predictive ability of the model. A Q^2^(Y) > 0.5 indicated good model quality.

^c^ a cross-validated ANOVA p-value < 0.05 indicated good model quality.

^d^ good permutation testing was achieved if Q^2^(Y) and R^2^(Y) values of the models based on the permutated data were significantly lower than those based on the real data set.

Using the validated OPLS-DA models, ions with discriminating power towards imposed treatments were selected for cows and calves, thereby implementing the strategy that was described in the section “Statistical analysis and data interpretation”. This enclosed the combined use of the S-plot (Fig 3), VIP-score, and Jack-knifed confidence intervals. As such, 76 positive and 21 negative ions were retained for calves, whereas 41 positive and 51 negative ions were selected for cows. These ions were retained as potential markers, including both indicators for untreated (most abundant in the controls) and TU treated (most abundant in the TU treated).

Following this, additional exclusions were made by considering sensitivity and specificity, calculated for each individual candidate marker. Hereto, threshold-values for sample classification according to TU origin were determined; i.e. a marker’s mean intensity (μ) plus or minus three times its standard deviation (σ) [32], as determined for the control group. Based on a minimum sensitivity and specificity of 80%, the number of candidate markers was reduced to 22 for calves and 53 for cows (S1 and S2 Tables). All of these compounds were found to be elevated under TU treatment, whereby some were considered true qualitative markers, meaning that their mere presence was able to point out the exogenous origin of detected TU, achieving the target value for sensitivity and specificity.

### Detection of candidate markers upon endogenous TU formation

Using the above-cited selection steps, candidate markers were determined that are able to differentiate between TU treated and untreated animals. However, to meet the principal objective of this study, these markers should also allow to discriminate between TU treated and untreated animals in which endogenous TU formation occurs. Therefore, some animals were administered a rapeseed-enriched diet in order to induce or promote endogenous formation of TU [9]. In this context, it should be noted that for the untreated control animals, receiving a conventional diet, low concentration levels of TU were already detected in urine; i.e. 6.1 ± 7.0 μg L^-1^ (n = 102; median of 2.48 μg L^-1^) for calves and 10.8 ± 11.7 μg L^-1^ (n = 102; median of 5.67 μg L^-1^) for cows. As such, the increase in urinary TU that was noted upon consumption of a rapeseed-enriched diet was rather limited and only significant for calves; i.e. an increase to 14.5 ± 5.8 μg L^-1^ (n = 35; median of 14.3 μg L^-1^; p-value of 8.7 e^-9^) for calves and to 12.7 ± 10.2 μg L^-1^ (n = 35; median of 9.19 μg L^-1^; p-value of 0.057) for cows (Mann-Whitney U, SPSS^®^ Statistics 23, IBM, USA). Nevertheless, additional candidate markers (i.e. 4 for the cows and 3 for the calves) could be excluded since sensitivity or specificity were below 80% when using thresholds (μ ± 3 σ) that were based on the data from those animals that were fed the rapeseed-enriched diet (S3 and S4 Tables).

### Metabolic linkage of candidate markers with urinary TU

To further endorse the relevance of each candidate marker, linkage between the urinary TU concentrations and marker levels was verified. Although no conclusions about a cause-and-effect relationship can be made, such a correlation study allowed to indicate metabolic events that are linked to other indirect effects of oral TU administration (e.g. stress responses). To this end, two equivalent strategies were considered; i.e. statistical correlation and OPLS modelling.

Kendall’s τ statistical correlations are presented in S3 and S4 Tables, whereby some candidate markers showed low correlation with urinary TU concentration (τ ranging from 0.135 to 0.454) and were therefore considered inferior. As an alternative tool, OPLS-models were established to describe the quantitative relationship between the urinary TU concentration and the metabolic fingerprints. In contrast to OPLS-DA models, the Y-variable is now a quantitative descriptor (TU concentration) instead of a qualitative classifier [34]. For both cows and calves, two valid OPLS-DA models were established; one for each ion polarity. These models were successfully validated by three model characteristics (R^2^(X) ≥ 0.497, R^2^(Y) ≥ 0.985, and Q^2^(Y) ≥ 0.727), permutation testing (n = 100), and CV-ANOVA (p-values ≤ 9.04 e^-10^). Subsequently, ions that were descriptive towards TU concentration were selected based on the Jack-knifed confidence interval (not across zero), VIP-score (≥ 1), and S-plot (corr(p) > 0.50, p ≤ -0.02 or ≥ 0.025 for calves, p ≤ -0.015 or ≥ 0.020 for cows). As such, additional candidate markers were excluded (S3 and S4 Tables). Eventually, based on this strategy, the number of markers was 12 for calves and 27 for cows.

### Tentative annotation of markers

Tentative identification of the remaining marker molecules was performed to consolidate their metabolic involvement in the exogenous administration of TU and extract unique compounds from isotope or adduct clusters. Hereto, the presence of ^13^C, ^33^S and S^34^ isotopes as well as the occurrence of multiple ionization adducts (i.e. [M+H]^+^, [M-H]^-^, and [M-H_2_O+H]^+^) was assorted. As such, with disregard of TU itself, in total, eleven unique compounds remained for cows and calves (S1 Fig). Chemical formula assignment and structural elucidation of these compounds was based on the accurate mass of the ^12^C structure (allowed deviations for formula matching ≤ 3 ppm), the ^13^C and ^34^S isotope profile, a number of heuristic rules [35], and the PRM-derived fragmentation spectrum (allowed deviations for *in silico* fragment matching ≤ 60 ppm or 0.02 Da). Data interpretation was supported by XCalibur 3.0 (Thermo Fisher Scientific, San Jose, USA), MZmine [35], CSI:FingerID [36], MetFrag [37], MAGMa, and MZCloud.

**Fig 4:**
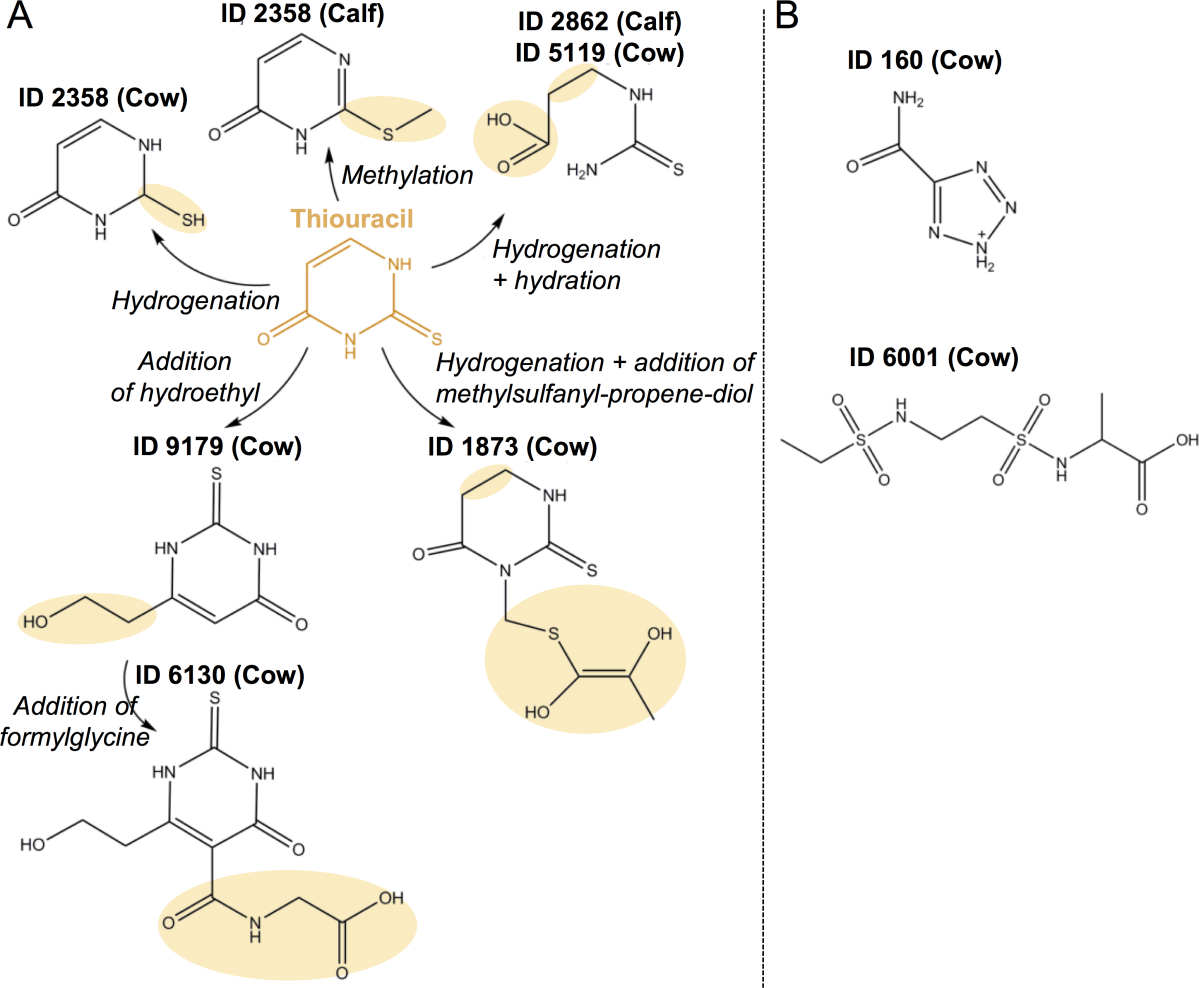
Chemical structures and metabolic linkage with TU for revealed biomarkers. The presented structures are those that were considered most likely according to *in silico* based and own interpretation of the fragmentation spectra. For the markers, depicted in figure panel A, a direct link with thiouracil could be determined, which was not case for the markers of panel B.

For a first set of compounds (Table 2, Fig 4), a strong structural relationship was noted between the proposed tentative identities and TU, which of course strongly supports the metabolic qualification of the respective markers. It can be assumed that the associated metabolization reactions, as suggested by Fig 4, take place in the liver [38]. However, it is remarkable that in case of endogenous TU, the suggested reactions were found to be less dominant or even lacking. Indeed, the urinary excretion of endogenous TU is indicative of an earlier presence in the blood stream and thus availability for the liver. The hypothesized explanation for this finding relates to the outcome of a recent study [10], by which it was demonstrated that endogenous formation of TU mainly takes place in the colonic part of the digestive system, with the presumption of an active microbial involvement. As such, the chemical or enzymatic modifications, inherent to the considered region in the gastro-intestinal tract (pre-colonic region for exogenous TU and colonic region in case of endogenous TU), may be decisive for the uptake by the liver or the metabolization reactions as mediated by the liver. Eventually, it should be noted that endogenous formation may take place in the rumen as well (although rather limited), which could explain the quantitative marker traits of ID 2385, ID 2835, ID 2862, and ID 5199.

**Table 2:** Biological markers that were considered descriptive towards TU treatment.

Compound ID (ionization mode)	Chemical formula	Isotope profile	PubChem ID	Fragmentation spectrum (structure matching fragments)
**Calves**				
2358 (+)	C_5_H_6_N_2_OS	5.2% ^13^C 4.4% ^34^S	ID number 79823 2-methylthiouracil	70.03, 71.03, 74.01, 95.02
2862 (+)	C_4_H_8_N_2_O_2_S	4.1% ^13^C 4.3% ^34^S	ID number 18341782 3-thioureidopropionic acid	55.02, 59.99, 72.04, 73.03, 77.02, 89.02, 90.06, 114.00, 131.03, 132.0
**Cows**				
2385 (+)	C_4_H_6_N_2_OS	4.0% ^13^C 3.0% ^34^S	ID number 15299396 2-sulfanyl-2,3-dihydro-1H-pyrimidin-4-one	55.02, 57.02, 69.02, 71.04, 84.04
5119 (+)	C_4_H_8_N_2_O_2_S	4.3% ^13^C 4.6% ^34^S	ID number 18341782 3-thioureidopropionic acid	55.02, 59.99, 72.04, 73.03, 77.02, 89.02, 90.06, 114.00, 131.03, 132.0
9179 (+)	C_6_H_8_N_2_O_2_S	8.8% ^13^C 3.1% ^34^S	ID number 21484601 6-(2-hydroxyethyl)-2-sulfanylidene-1H-pyrimidin-4-one	55.04, 67.04, 69.06, 71.05, 85.05, 95.05,113.05
160 (-)	C_2_H_4_ON_5_	4.1% ^13^C 0% ^34^S	ID number 3396861 2H-tetrazol-2-ium-5-carboxamide	68.99, 69.03, 70.00, 70.02, 96.01
1873 (-)^*a*^	C_8_H_12_O_3_N_2_S_2_	7.6% ^13^C 8.2% ^34^S	ID number 17446869 3-(1,1-dioxo-3-thiolanyl)-5-methyl-2-thioxo-4-imidazolidinone	57.03, 58.98, 121.03, 129.01, 135.03
6001 (-)	C_7_H_16_O_6_N_2_S_2_	6.7% ^13^C 8.2% ^34^S	ID number 54274647 (ChemSpider) N-({2-[(ethylsulfonyl) amino]ethyl}sulfonyl)-L-alanine	108.04, 139.01, 188.00
6130 (-)^*a*^	C_9_H_15_O_6_N_3_S	7.4% ^13^C 3.7% ^34^S	ID number 18219810 3-[(2-amino-3-sulfanylpropanoyl) amino]-4-(carboxymethylamino)-4-oxobutanoic acid	72.01, 100.00, 117.01, 118.02, 139.01, 141.01, 156.03, 172.05, 173.03

Identification was performed upon the level of putatively annotated compound classes [39]. The data include the identities as they were determined by an *in silico* based approach.

^a^ Tentative identities as determined by *in silico*-based interpretation of fragmentation data. Starting from these matching fragments, other chemical configurations were sometimes considered more likely in light of the TU context and presented in Fig 4. It was effectively verified that these structures were not incorporated in online databases and therefore could not be suggested by the *in silico*-based approach.

Whereas the suggested identities for ID 2358, ID 2385, ID 2862, and ID 5199 were retrieved by an *in silico* based approach (using online databases such as ChemSpider and PubChem), the structural configuration of ID 1873 and ID 6130 were established otherwise. *In silico* based interpretation of the fragmentation data of ID 6130 pointed towards a monocarboxylic acid in which a sulphur group was present (i.e. PubChem ID 18219810, Table 2). As such, metabolic qualification of this marker could be presumed although the linkage with TU would involve a relatively complex metabolization pathway. However, on the basis of the matching fragments, another chemical configuration could be proposed, whereby the ring substructure could be substituted by a TU-related configuration (Fig 4). The strong resemblance with ID 9179 reinforced this finding. This structure was not included in none of the consulted databases (ChemSpider, KEGG, HMDB and PubChem) and was therefore not pointed out by the *in silico* based strategy. Also for ID 1873, own interpretation of the fragments that matched the *in silico* based structure pointed towards thiouracil as a more likely substructure of the chemical skeleton (Fig 4). Here, some typical fragments of TU (i.e. 70.02 and 111.99) were also found in the fragmentation pattern of ID 1873. Again, when applying a targeted screening in online available databases, it was not able to retrieve the suggested structure. Interestingly, one of the marker molecules that was determined for cows (ID 5119) was also revealed as biomarker for calves (ID 2862). For this marker, the suggested identity was again strongly linked with TU for which a hydrogenation and hydration reaction were determined. Taking into account the fact that the retention time of this marker is similar to that of TU, it was verified and confirmed that it was not an alternative ionization product of TU.

For the other compounds, i.e. ID 160 and ID 6001, no direct link with TU could be made. To the best of our knowledge, no studies have reported on these compounds. In addition, pathway analysis software tools such as MetaboAnalyst 3.0 [40] and Small Molecule Pathway Database [41] did not enclose any information on these compounds as well.

Unfortunately, analytical reference standards were not available for all of the above-suggested structures, precluding additional confirmatory identification actions. For ID 7334, 7552, and ID 7676, no fragmentation data could be obtained as compound degradation occurred during the period between sample full-scan analysis and targeted PRM fragmentation. For these compounds, identification initiatives only enclosed chemical formula matching, pointing towards C_5_H_13_O_8_N_9_ for ID 7334, C_5_H_18_O_8_N_9_ for ID 7552, and C_10_H_14_O_6_N_3_S_2_ for ID 7676.

### Performance of biomarker panels during ongoing TU treatment

The performance of the selected markers to appoint the true origin of detected TU was evaluated by considering integrative biomarker panels. To this end, weighted equations (Eqs 1 and 2) were introduced to generate a mathematical descriptor (Y), indicative for the biomarker panel and characteristic for each sample. These equations are function of the markers’ abundance and presented below.

$$\begin{array}{l} Ycows=0.061\;log\;\left[ID\;160\right]+0.065\;log\;[ID\;1873]\;+\;0.061\;log\;\left[ID\;9179\right]+0.049\;log\;\left[ID\;6001\right]+\\ 0.057\;log\;\left[ID\;6130\right]+0.058\;log\;\left[ID\;7334\right]+0.058\;log\;\left[ID\;7552\right]+0.061\;log\;\left[ID\;7676\right]+\\ 0.038\;log\;\left[ID\;2385\right]+0.033\;log\;\left[ID\;5199\right]+0.028\;log\;\left[TU\right] \end{array}$$ (Eq 1)

Ycalves=0.517log[ID2385]+0.332log[ID2862]−0.04log[TU] (Eq 2)

Using the Y-values of the samples with endogenous TU (rapeseed-enriched diet and control group), predictive screening criteria were determined, being calculated as the average (μ) plus two (95^th^ percentile of confidence) or three times (99^th^ percentile of confidence) the standard deviation (σ) [32].

**Fig 5:**
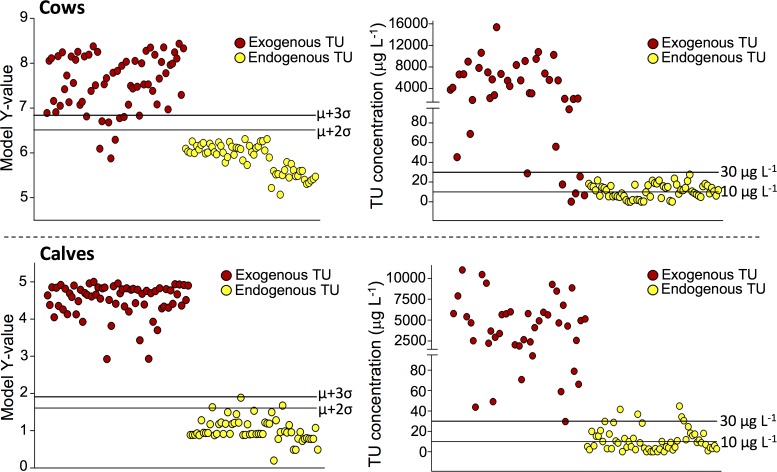
Sample classification plots with the model Y-value or TU concentration as classifying variable. Associated threshold values are indicated by the horizontal lines. Hereby, a correct assignment of TU origin (having or not having an exogenous component) is intended.

For cows, a screening criterion of 1.61 and 1.91 was determined for the 95^th^ and 99^th^ percentile of confidence, respectively. Implementation of these criteria for classification of samples with exogenous TU (coming from TU-treated animals) and endogenous TU (coming from the rapeseed-enriched diet and control group) rendered sensitivity values of 100% and specificity values of 94.9% (95^th^ percentile of confidence) or 100% (99^th^ percentile of confidence) (Fig 5). In this context, it may be noted that the OPLS-DA model, used for generation of the mathematical equation, was not assumed to be over-fitted as evidenced by the validation output (p-value of 7.2 e^-34^ and Q^2^(Y) of 0.746). Nevertheless, the expediency of the multi-biomarker approach was also verified by keeping approximately 1/7^th^ of the samples out of the modelling dataset and using them as an external dataset. After calculation of the screening criteria (1.56 for the 95^th^ percentile of confidence and 1.89 for the 99^th^ percentile of confidence), this yielded a sensitivity (n = 9) of 100% and specificity (n = 9) of 88.9% for both percentiles. As these findings are in line with the previously-mentioned ones, the robustness of the used OPLS-DA model and the associated screening criteria was affirmed. Moreover, when the classification scores were compared with those that were obtained using TU-based thresholds [17], the applied multi-biomarker approach was found to perform better. Indeed, considering a TU-threshold of 10 μg L^-1^, sensitivity and specificity values of 100% and 55.0% were achieved, respectively, whereas this was 98.4% and 86.7% in case of a 30 μg L^-1^ threshold (Fig 5).

A same strategy was applied for the calves, using a screening criterion of 6.51 and 6.83 for the 95^th^ and 99^th^ percentile of confidence, respectively. These criteria resulted in sensitivity values of 95.2 and 85.7%, while specificities of 100% were achieved (Fig 5). Robustness of the model was verified based on the validation data (p-value < 0.001 and Q^2^(Y) of 0.951) and use of an external dataset, again generated by randomly removing 1/7^th^ of the samples from the entire dataset. This yielded similar classification scores as before, i.e. sensitivities (n = 9) of 88.9% and a specificity (n = 9) of 88.9% or 100%. Comparison with the TU-based classification scores (a sensitivity of 90.8% and specificity of 100%) indicated a better performance of the multi-marker approach in case that the 95^th^ percentile of confidence screening criterion was implemented. Consideration of the 10 μg L^-1^ threshold was not of any value as unacceptable results were obtained for sample classification (sensitivity and specificity ≤ 53.8%) (Fig 5).

Based on the listed classification scores, it may be concluded that the presented biomarker panels may assist in differentiating between samples with endogenous and exogenous TU, with the latter originating from ongoing treatment. This statement is especially true for the cows, where the relatively high number of marker molecules contributes towards excellent predictability.

### Performance of biomarker panels at low exogenous TU levels

Next to the classification performance of the biomarker panels in case of ongoing TU treatment, the markers’ performance in those situations in which low levels of exogenous TU are more likely is also relevant, i.e. in case of a recently ended treatment or when TU is administered as a low-dose drug cocktail.

**Fig 6:**
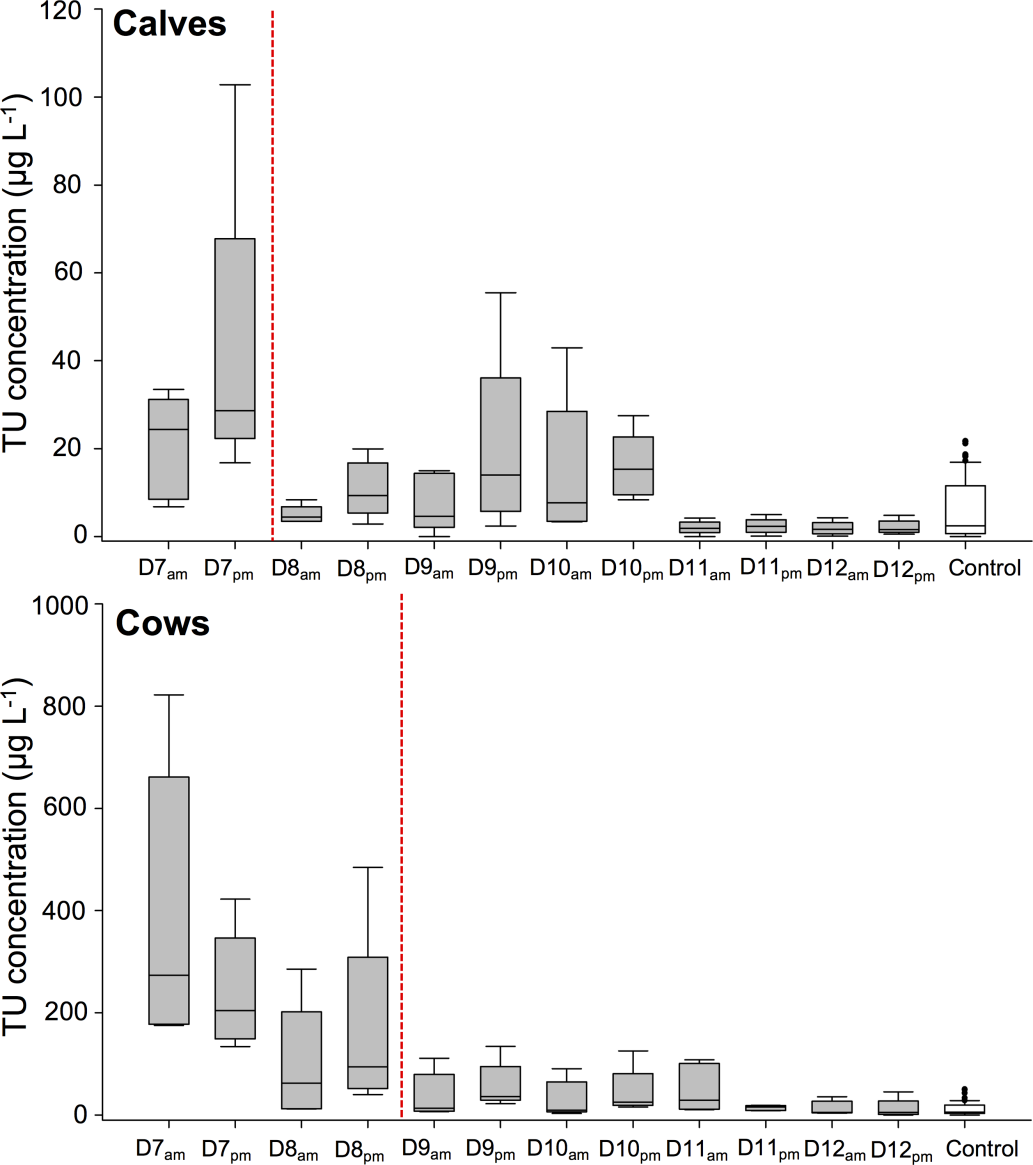
Boxplots of TU concentration profiles. These data are presented for the calves and adult cows during the washout period. Data were obtained from 5 animals. The red dashed line indicates the first sampling moment after last TU administration for which the measured TU concentration was no longer statistically different from the control group. Last TU administration took place at D6 am.

In first instance, the washout period was considered, whereby a gradual decline in urinary exogenous TU concentration can be observed (Fig 6). However, TU free urine was never reached because of the occurrence of semi-endogenous TU formation. In order to estimate the time by which exogenous TU had probably disappeared, total TU concentrations were for each time-point of the washout period statistically evaluated against the endogenous concentrations of the control groups. For calves, exogenous TU was estimated to have washed out at 48 h after last administration, whereas this was 72 h for the adult cows (SPSS^®^ Statistics 23, Mann-Whitney U, p-value > 0.05). Considering these assumed excretion profiles for exogenous TU, a high sensitivity for the biomarker panels along the various time-points has of course most potential to reveal a past administration of synthetic TU.

**Fig 7:**
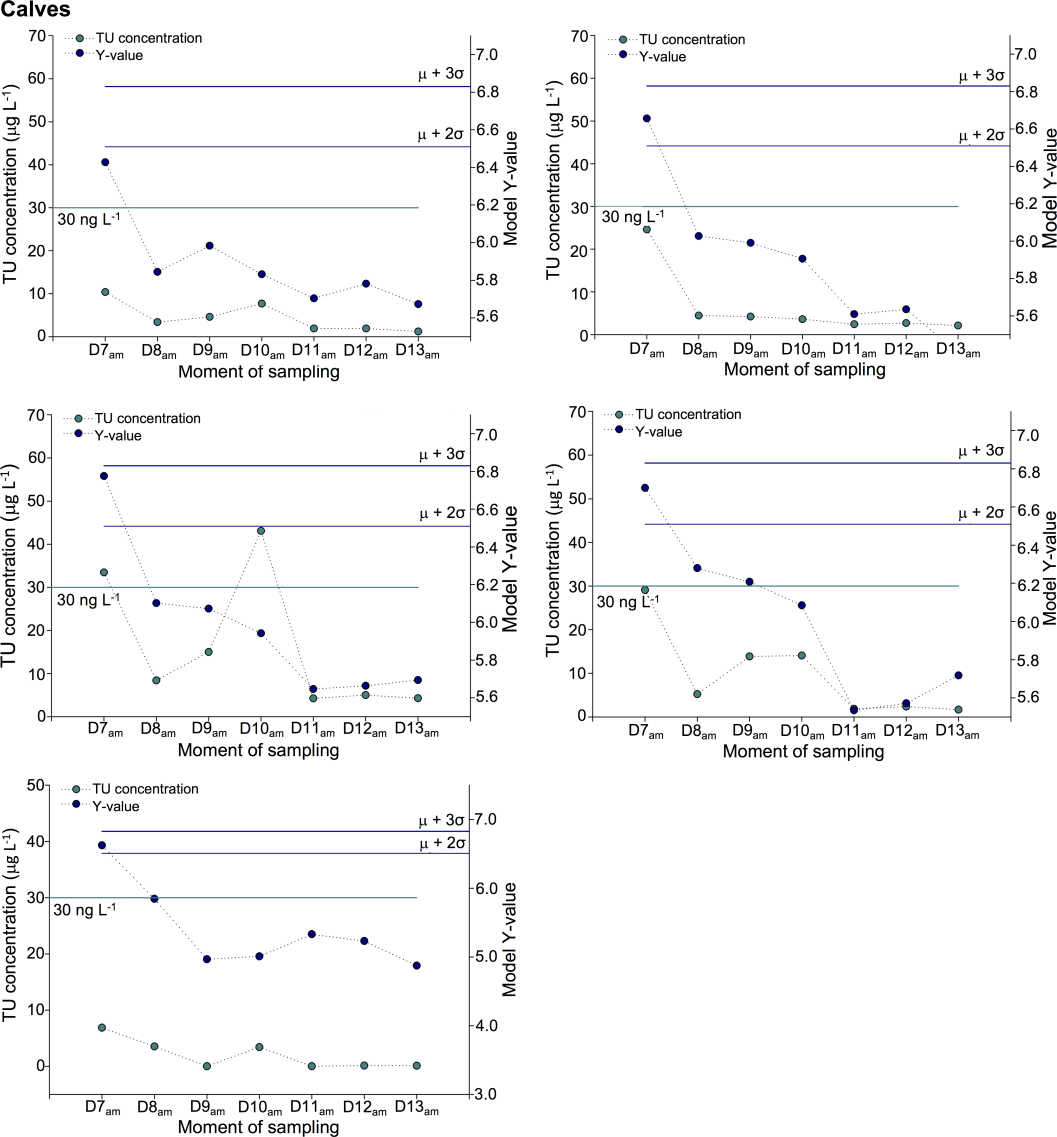
TU excretion profiles for calves. Comparison of TU concentration levels and biomarker profiles in their capacity to indicate past TU treatment (last administration took place at D6am). For each classification strategy, the associated threshold values are presented.

For the calves, it was possible to indicate a recent TU treatment for one out five calves until 24 h after the last administration, implementing the 30 ng μL^-1^ threshold (Fig 7). These results indicate a fast excretion of exogenous TU with a sharp decline in total urinary TU concentration. Using the biomarker panel and the 95^th^ percentile of confidence screening criterion, the TU treatment was demonstrable for four calves and this until 24 h. As such, a more sensitive monitoring of TU abuse is concluded for the multi-marker approach relative to the single TU marker.

**Fig 8:**
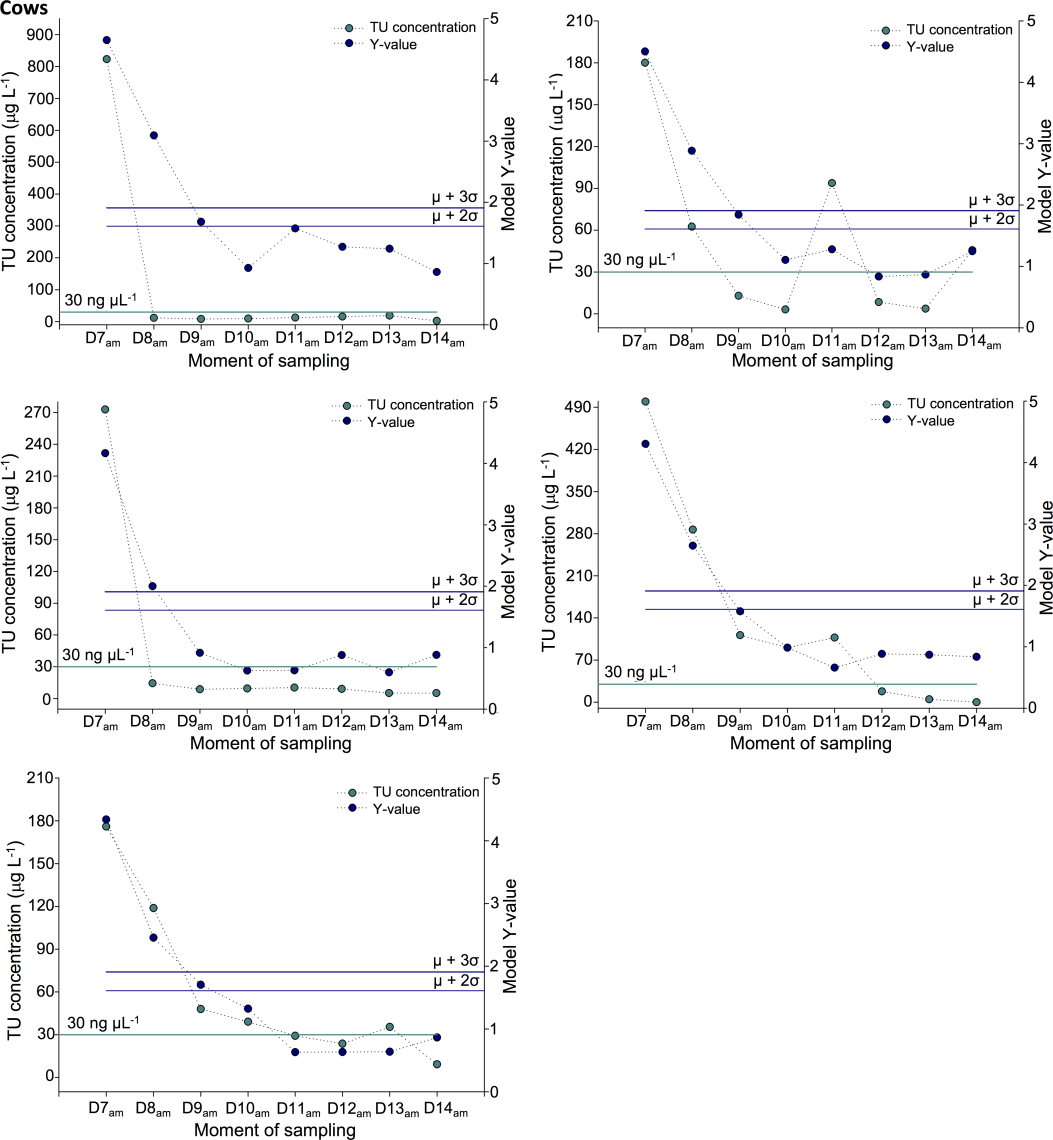
TU excretion profiles for cows. Comparison of TU concentration levels and biomarker profiles in their capacity to indicate past TU treatment (last administration took place at D6am). For each classification strategy, the associated threshold values are presented.

TU excretion profiles for the cows were more diverse in comparison with calves, whereby for some of the test animals a rather slow decline in exogenous TU concentration was observed along the washout period (Fig 8). These slower excretion rates gave rise to a longer detection of TU treatment, using the 30 μg L^-1^ TU-threshold. Indeed, the past treatment could be demonstrated until 24 h after last TU administration for all cows, until 48 h for three cows and until 72 h for two cows. Using the multi-marker approach, TU treatment could be ascertained for all cows until 48 h on the basis of the 99^th^ percentile of confidence screening criterion (Fig 8). Moreover, treatment was demonstrable for four cows until 72 h in case that the 95^th^ percentile of confidence criterion was used. This allowed to conclude a more reliable detection for the first phase of the washout period, whereby it should be noted that for those cows with a strong TU persistency (above the 30 μg L^-1^ threshold until 96 h), the biomarker panel was not able to capture this.

Finally, a dilution experiment was conducted to simulate a low-dose TU treatment and estimate the lower limits of TU for which the biomarker panel is still able to indicate its exogenous origin. More specifically, the markers’ detectability at low exogenous TU levels was effectuated by diluting pooled extracts (either for morning or evening urine) from TU treated animals (n = 3, distinguishing between cows and calves). Pooled morning and pooled evening urine extracts were diluted 1/10, 1/25, 1/50, and 1/100. Hereby, the pooled extracts from three control animals was used as dilution solvent, allowing to reach low exogenous TU levels while potential matrix influences and endogenous TU were preserved. With this strategy, which was based on the significant statistical correlations between exogenous TU concentrations and marker intensities (Kendall’s τ from 0.507 to 0.986, p-value ≤ 0.05), the screening criterions were again used to determine the dilution factor for which a sample would still be classified as containing exogenous TU. For calves, exogenous TU could still be indicated for a total TU concentration of 16.9 μg L^-1^ (on the basis of the 95^th^ percentile of confidence), whereas this was 2.0 μg L^-1^ (on the basis of the 95^th^ percentile of confidence) for the cows. These results indicate that ongoing low-dose TU treatment can be revealed using our biomarker panel strategy, even when urinary concentrations of exogenous TU are below the currently suggested threshold of 30 μg L^-1^.

## Supporting information

S1 Text Tested extraction protocols. Protocols that were tested for the efficient extraction and high metabolome coverage of metabolites from urine, thereby having a particular focus on thyreostats.
(DOCX)

S1 Table Ions that were retained as candidate markers for calves. Ions were able to discriminate between TU treated and untreated calves. All ions showed the highest abundance upon TU treatment.
(DOCX)

S2 Table Ions that were retained as candidate markers for cows. Ions were able to discriminate between TU treated and untreated cows. All ions showed the highest abundance upon TU treatment.
(DOCX)

S3 Table Filtering of candidate markers for calves. Selection of candidate markers based on the sensitivity and specificity as determined for the TU treated calves and those that received the rapeseed-enriched diet. In addition, based on metabolic linkage (correlation coefficient and OPLS modelling), additional certainty about the metabolic involvement of the markers with respect to TU treatment was obtained.
(DOCX)

S4 Table Filtering of candidate markers for cows. Selection of candidate markers based on the sensitivity and specificity as determined for the TU treated cows and those that received the rapeseed-enriched diet. In addition, based on metabolic linkage (correlation coefficient and modelling), additional certainty about the metabolic involvement of the markers with respect to TU treatment was obtained.
(DOCX)

S1 Fig Chromatograms for the eleven metabolite markers. Chromatograms were acquired from samples from TU treated animals, with characteristic information about the retention time (RT), *m/z*-value, ionization adduct, intensity, and tentative chemical configuration.
(DOCX)
