# Supplementary material for: Discovery of urinary biomarkers to discriminate between exogenous and semi-endogenous thiouracil in cattle: A parallel-like randomized design
Source: PLoS One. 2018 Apr 12;13(4):e0195351. doi: 10.1371/journal.pone.0195351 (PMC5896977; doi:10.1371/journal.pone.0195351)
Supplement: S1 Text — Protocols that were tested for the efficient extraction and high metabolome coverage of metabolites from urine, thereby having a particular focus on thyreostats. (DOCX) [file pone.0195351.s001.docx]

Discovery of Urinary Biomarkers to Discriminate Between Exogenous and Semi-Endogenous Thiouracil in Cattle: A Parallel-Like Randomized Design

Thiouracil administration in cattle and urinary biomarkers

Lieven Van Meulebroek^a^, Jella Wauters^a^, Beata Pomian^a^, Julie Vanden Bussche^a^, Philippe Delahaut^b^, Eric Fichant^b^, Lynn Vanhaecke^a^

^a^ Ghent University, Faculty of Veterinary Medicine, Department of Veterinary Public Health and Food Safety, Laboratory of Chemical Analysis, Salisburylaan 133, 9820 Merelbeke, Belgium;

^b^ CER Groupe, Health Department, Rue Point du Jour 8, 6900 Marloie, Belgium.

**S1 Text.** **Tested extraction protocols.** Protocols that were tested for the efficient extraction and high metabolome coverage of metabolites from urine, thereby having a particular focus on thyreostats.

**Consulted references**

C. C. Jacob, G. Dervilly-Pinel, G. Biancotto, F. Monteau, B. Le Bizec, *Metabolomics*, 2015, 11, 184-197.

G. Dervilly-Pinel, S. Chereau, N. Cesbron, F. Monteau, B. Le Bizec, 2015, *Metabolomics*, 2015, 11, 403-411.

R. Yue, L. Zhao, Y. Hu, P. Jiang, S. Wang, L. Xiang, W. Liu, W. Zhang, R. Liu, *J Ethnopharmacol,* 2013, 2, 465-475.

C. H. Johnson, S. K. Manna, K. W. Krausz, J. A. Bonzo, R. D. Divelbiss, M. G. Hollingshead, F. J. Gonzalez, *Metabolites*, 2013, 3, 658-672.

J. A. L. Kiebooms, J. Wauters, J. Vanden Bussche, L. Vanhaecke, *J Chrom A*, 1345, 164-173.

H. Wen, T. Lee, S. You, S. Park, H. Song, K. S. Eilber, J. T. Anger, M. R. Freeman, S. Park, J. Kim, J Proteome Res, 2015, 14, 541-548.

J. Vanden Bussche, L. Vanhaecke, Y. Deceuninck, K. Verheyden, K. Wille, K. Bekaert, B. Le Bizec, H. F. De Brabander, *Journal of Chromatography A*, 2010, 1217, 4285-4293.

**Extraction protocols**

*Protocol 1:* 500 μL of cottonwool-filtered urine was purified using a 10 kDa Amicon Ultra centrifugal unit (Merck Millipore Corporation, Massachusetts, USA) (10,000 g at 5 °C for 20 min). 10 μL of the purified extract was injected.

*Protocol 2:* 500 μL of cottonwool-filtered urine was purified by using a 30 kDa Amicon Ultra centrifugal unit (Merck Millipore Corporation, Massachusetts, USA) (10,000 g at 5 °C for 20 min). 10 μL of the purified extract was injected.

*Protocol 3:* 200 μL methanol was added to 200 μL of cottonwool-filtered urine and vortexed for 1 min. Afterwards, the solution was centrifuged at 12,000 rpm for 10 min and at 5 °C. The supernatants was collected into a LC-vial and 10 μL was injected.

*Protocol 4:* 200 μL acetonitrile/ultrapure water mixture (50/50, v/v) (acidified with 5 μM chlorpropamide) was added to 200 μL cottonwool-filtered urine and vortexed for 1 min. Afterwards, the solution was centrifuged at 14,000 rpm for 20 min and at 4 °C. The supernatants was collected into a LC-vial and 10 μL was injected.

*Protocol 5:* 500 μL of cottonwool-filtered urine was purified using a 30 kDa Amicon Ultra centrifugal unit (Merck Millipore Corporation, Massachusetts, USA) (15,000 g at 5 °C for 30 min). Afterwards, the extract was purified by solid phase extraction with Oasis HLB cartridges (60 mg, 3 cc, Waters GmbH, Germany). The extract was evaporated to dryness and dissolved in 500 μL mobile phase. 10 μL of this solution was injected.

*Protocol 6:* 500 μL of cottonwool-filtered urine was purified by centrifugation only (3000 g at 5 °C for 10 min). 10 μL of the extract was injected.

*Protocol 7:* 500 μL of DTT-enriched (1%) phosphate buffer (pH 7) was added to 500 μL of cottonwool-filtered urine and vortexed. Samples were placed under danaturation conditions (65 °C for 30 min), after which a two-fold liquid-liquid extraction with 2.5 mL ethyl acetate took place. The combined extracts were evaporated to dryness and dissolved in 500 μL mobile phase. 10 μL of this solution was injected.
