# Supplementary material for: Discovery of urinary biomarkers to discriminate between exogenous and semi-endogenous thiouracil in cattle: A parallel-like randomized design
Source: PLoS One. 2018 Apr 12;13(4):e0195351. doi: 10.1371/journal.pone.0195351 (PMC5896977; doi:10.1371/journal.pone.0195351)
Supplement: S1 Table — Ions were able to discriminate between TU treated and untreated calves. All ions showed the highest abundance upon TU treatment. (DOCX) [file pone.0195351.s002.docx]

Discovery of Urinary Biomarkers to Discriminate Between Exogenous and Semi-Endogenous Thiouracil in Cattle: A Parallel-Like Randomized Design

Thiouracil administration in cattle and urinary biomarkers

Lieven Van Meulebroek^a^, Jella Wauters^a^, Beata Pomian^a^, Julie Vanden Bussche^a^, Philippe Delahaut^b^, Eric Fichant^b^, Lynn Vanhaecke^a^

^a^ Ghent University, Faculty of Veterinary Medicine, Department of Veterinary Public Health and Food Safety, Laboratory of Chemical Analysis, Salisburylaan 133, 9820 Merelbeke, Belgium;

^b^ CER Groupe, Health Department, Rue Point du Jour 8, 6900 Marloie, Belgium.

**S1 Table.** **Ions that were retained as candidate markers for calves**.

| **compound ID** | **ioniz. mode** | ***m/z* (Da)** | **retention time (min)** | **sensitivity (%)**  **(n = 64)** | **specificity (%)**  **(n = 39)** |
| --- | --- | --- | --- | --- | --- |
| 1683 | - | 147.0223 | 1.59 | 90.6 | 94.9 |
| 1686*^a^* | - | 244.9906 | 1.59 | 85.9 | 100 |
| 8189 | - | 307.1587 | 8.73 | 96.9 | 100 |
| 358*^a^* | + | 111.9855 | 1.53 | 88.9 | 100 |
| 360 | + | 130.0151 | 1.53 | 95.2 | 94.9 |
| 361 | + | 131.0076 | 1.53 | 95.2 | 97.4 |
| 2358 | + | 143.0276 | 3.91 | 98.4 | 100 |
| 2425 | + | 144.0271 | 4.14 | 85.7 | 97.4 |
| 2427 | + | 144.0305 | 4.13 | 93.7 | 97.4 |
| 2497 | + | 145.0230 | 4.14 | 93.7 | 100 |
| 2862 | + | 149.0379 | 1.58 | 90.4 | 100 |
| 2906 | + | 150.0374 | 1.56 | 84.1 | 97.4 |
| 2908*^a^* | + | 150.0410 | 1.55 | 92.1 | 97.4 |
| 2978*^a^* | + | 151.0336 | 1.56 | 93.7 | 97.4 |
| 4979 | + | 173.0380 | 4.98 | 84.1 | 100 |
| 4981*^a^* | + | 173.0381 | 6.44 | 88.9 | 97.4 |
| 10417 | + | 269.0624 | 1.53 | 82.5 | 100 |
| 12414 | + | 249.0364 | 4.53 | 84.1 | 100 |
| 13485*^a^* | + | 262.0314 | 6.16 | 81.0 | 100 |
| 13657 | + | 264.0799 | 6.00 | 88.9 | 94.9 |
| 16641 | + | 322.0967 | 6.19 | 87.3 | 97.4 |
| 17771 | + | 323.0998 | 6.32 | 85.7 | 97.4 |

Ions were able to discriminate between TU treated and untreated calves. All ions showed the highest abundance upon TU treatment. ^a^ So-called qualitative candidate markers, whereby sensitivity and specificity values of ≥ 80% were also reached when a threshold of zero was taken into consideration.
