# Supplementary material for: Discovery of urinary biomarkers to discriminate between exogenous and semi-endogenous thiouracil in cattle: A parallel-like randomized design
Source: PLoS One. 2018 Apr 12;13(4):e0195351. doi: 10.1371/journal.pone.0195351 (PMC5896977; doi:10.1371/journal.pone.0195351)
Supplement: S2 Table — Ions were able to discriminate between TU treated and untreated cows. All ions showed the highest abundance upon TU treatment. (DOCX) [file pone.0195351.s003.docx]

Discovery of Urinary Biomarkers to Discriminate Between Exogenous and Semi-Endogenous Thiouracil in Cattle: A Parallel-Like Randomized Design

Thiouracil administration in cattle and urinary biomarkers

Lieven Van Meulebroek^a^, Jella Wauters^a^, Beata Pomian^a^, Julie Vanden Bussche^a^, Philippe Delahaut^b^, Eric Fichant^b^, Lynn Vanhaecke^a^

^a^ Ghent University, Faculty of Veterinary Medicine, Department of Veterinary Public Health and Food Safety, Laboratory of Chemical Analysis, Salisburylaan 133, 9820 Merelbeke, Belgium;

^b^ CER Groupe, Health Department, Rue Point du Jour 8, 6900 Marloie, Belgium.

**S2 Table. Ions that were retained as candidate markers for cows.**

| **compound ID** | **ioniz. mode** | ***m/z* (Da)** | **retention time (min)** | **sensitivity (%)**  **(n = 65)** | **specificity (%)**  **(n = 38)** |
| --- | --- | --- | --- | --- | --- |
| 160*^a^* | - | 113.0343 | 1.58 | 100 | 100 |
| 405 | - | 126.9962 | 1.54 | 87.7 | 100 |
| 406 | - | 126.9959 | 2.26 | 84.6 | 100 |
| 445*^a^* | - | 127.9953 | 1.55 | 86.2 | 100 |
| 457*^a^* | - | 128.9918 | 1.54 | 100 | 94.7 |
| 875*^a^* | - | 147.0226 | 1.90 | 86.5 | 97.4 |
| 920^a^ | - | 149.0181 | 1.52 | 98.5 | 100 |
| 1873*^a^* | - | 247.0211 | 5.06 | 100 | 97.4 |
| 6001^a^ | - | 287.0377 | 1.62 | 100 | 97.4 |
| 6130*^a^* | - | 292.0611 | 1.62 | 100 | 97.4 |
| 7106*^a^* | - | 319.0719 | 1.58 | 90.8 | 94.7 |
| 7334*^a^* | - | 326.0819 | 1.59 | 93.8 | 100 |
| 7552*^a^* | - | 332.1288 | 1.57 | 93.8 | 100 |
| 7676*^a^* | - | 336.0332 | 1.61 | 98.5 | 97.4 |
| 11934 | - | 483.0589 | 7.54 | 89.2 | 97.4 |
| 466*^a^* | + | 111.9854 | 1.53 | 96.9 | 92.3 |
| 467*^a^* | + | 131.0076 | 1.53 | 100 | 100 |
| 468*^a^* | + | 130.0151 | 1.53 | 100 | 100 |
| 2385 | + | 131.0275 | 1.30 | 98.5 | 97.4 |
| 2386*^a^* | + | 133.0232 | 1.30 | 83.1 | 97.4 |
| 4357*^a^* | + | 144.0246 | 2.94 | 100 | 100 |
| 4359*^a^* | + | 144.0270 | 2.95 | 96.9 | 97.4 |
| 4361 | + | 144.0306 | 2.95 | 100 | 97.4 |
| 4466 | + | 145.0231 | 2.94 | 98.5 | 97.4 |
| 5119 | + | 149.0379 | 1.50 | 100 | 97.4 |
| 5120 | + | 150.0373 | 1.50 | 100 | 97.4 |
| 6145*^a^* | + | 157.0391 | 6.14 | 86.2 | 94.9 |
| 6152*^a^* | + | 157.0432 | 5.15 | 95.4 | 94.9 |
| 9176 | + | 173.0380 | 3.71 | 98.5 | 97.4 |
| 9177 | + | 173.0381 | 4.72 | 100 | 97.4 |
| 9179*^a^* | + | 173.0380 | 6.53 | 98.5 | 97.4 |
| 16756*^a^* | + | 216.0437 | 2.26 | 100 | 97.4 |
| 20949 | + | 249.0360 | 3.44 | 100 | 97.4 |
| 20952 | + | 249.0359 | 4.60 | 98.5 | 97.4 |
| 20954 | + | 251.0312 | 4.60 | 98.5 | 97.4 |
| 21117*^a^* | + | 251.0511 | 1.34 | 96.9 | 94.9 |
| 21221*^a^* | + | 252.0358 | 1.38 | 100 | 94.9 |
| 22297*^a^* | + | 262.0313 | 6.11 | 96.9 | 100 |
| 22303*^a^* | + | 262.0643 | 6.18 | 92.3 | 100 |
| 22481 | + | 264.0800 | 5.99 | 100 | 97.4 |
| 22670*^a^* | + | 266.0757 | 6.00 | 96.9 | 100 |
| 22933 | + | 269.0618 | 1.46 | 100 | 97.4 |
| 23815 | + | 278.0951 | 6.28 | 92.3 | 100 |
| 23951 | + | 280.0747 | 5.18 | 96.9 | 97.4 |
| 24312 | + | 283.0772 | 2.97 | 95.4 | 97.4 |
| 26256*^a^* | + | 305.0689 | 6.20 | 90.8 | 97.4 |
| 26257 | + | 322.0966 | 6.20 | 98.5 | 94.9 |
| 27655 | + | 324.0924 | 6.20 | 95.4 | 97.4 |
| 28490 | + | 336.1114 | 6.36 | 87.7 | 94.9 |
| 29853 | + | 434.1467 | 6.28 | 93.8 | 97.4 |
| 30488 | + | 364.1422 | 6.39 | 96.9 | 97.4 |
| 32967 | + | 408.1316 | 6.11 | 93.8 | 97.4 |
| 33682 | + | 422.1470 | 6.21 | 96.9 | 97.4 |

Ions were able to discriminate between TU treated and untreated cows. All ions showed the highest abundance upon TU treatment. ^a^ So-called qualitative candidate markers, whereby sensitivity and specificity values of ≥ 80% were also reached when a threshold of zero was taken into consideration.
