# Supplementary material for: Discovery of urinary biomarkers to discriminate between exogenous and semi-endogenous thiouracil in cattle: A parallel-like randomized design
Source: PLoS One. 2018 Apr 12;13(4):e0195351. doi: 10.1371/journal.pone.0195351 (PMC5896977; doi:10.1371/journal.pone.0195351)
Supplement: S3 Table — Selection of candidate markers based on the sensitivity and specificity as determined for the TU treated calves and those that received the rapeseed-enriched diet. In addition, based on metabolic linkage (correlation coefficient and OPLS modelling), additional certainty about the metabolic involvement of the markers with respect to TU treatment was obtained. (DOCX) [file pone.0195351.s004.docx]

Discovery of Urinary Biomarkers to Discriminate Between Exogenous and Semi-Endogenous Thiouracil in Cattle: A Parallel-Like Randomized Design

Thiouracil administration in cattle and urinary biomarkers

Lieven Van Meulebroek^a^, Jella Wauters^a^, Beata Pomian^a^, Julie Vanden Bussche^a^, Philippe Delahaut^b^, Eric Fichant^b^, Lynn Vanhaecke^a^

^a^ Ghent University, Faculty of Veterinary Medicine, Department of Veterinary Public Health and Food Safety, Laboratory of Chemical Analysis, Salisburylaan 133, 9820 Merelbeke, Belgium;

^b^ CER Groupe, Health Department, Rue Point du Jour 8, 6900 Marloie, Belgium.

**S3 Table. Filtering of candidate markers for calves.**

| **compound ID** | **sensitivity**  **(%) (n = 64)** | **specificity  (%) (n = 21)** | **correlation coefficient (τ)** | **OPLS-based selection** |
| --- | --- | --- | --- | --- |
| 358 | 87.3 | 95.2 | 0.861 | ✓ |
| 360 | 96.8 | 100 | 0.866 | ✓ |
| 361 | 87.3 | 95.2 | 0.864 | ✓ |
| 2358 | 100 | 95.2 | 0.547 | ✓ |
| 2425 | 87.3 | 95.2 | 0.507 | ✓ |
| 2427 | 93.7 | 100 | 0.521 | ✓ |
| 2497 | 93.7 | 100 | 0.516 | ✓ |
| 2862 | 87.3 | 100 | 0.525 | ✓ |
| 2906 | 84.1 | 100 | 0.515 | ✓ |
| 2908 | 88.9 | 95.2 | 0.517 | ✓ |
| 2978^b^ | 88.9 | 95.2 | 0.513 | ✓ |
| 4979 | 90.5 | 95.2 | **0.245** | **✗** |
| 4981 | 88.9 | 100 | **0.436** | **✗** |
| 10417 | 84.1 | 95.2 | **0.320** | **✗** |
| 12414 | 98.4 | 95.2 | **0.377** | **✗** |
| 13485 | 81.0 | 100 | **0.360** | **✗** |
| 13657 | 85.7 | 95.2 | **0.454** | ✓ |
| 16641 | 96.8 | 95.2 | **0.230** | **✗** |
| 17771 | 95.3 | 95.2 | **0.232** | **✗** |
| 1683 | 85.9 | 100 | 0.529 | ✓ |
| 1686 | **73.4** | 100 | **0.462** | ✓ |
| 8189 | **21.9** | **95.2** | **0.330** | **✗** |

Selection of candidate markers based on the sensitivity and specificity as determined for the TU treated calves and those that received the rapeseed-enriched diet. In addition, based on metabolic linkage (correlation coefficient and OPLS modelling), additional certainty about the metabolic involvement of the markers with respect to TU treatment was obtained.
