# Supplementary material for: Discovery of urinary biomarkers to discriminate between exogenous and semi-endogenous thiouracil in cattle: A parallel-like randomized design
Source: PLoS One. 2018 Apr 12;13(4):e0195351. doi: 10.1371/journal.pone.0195351 (PMC5896977; doi:10.1371/journal.pone.0195351)
Supplement: S4 Table — Selection of candidate markers based on the sensitivity and specificity as determined for the TU treated cows and those that received the rapeseed-enriched diet. In addition, based on metabolic linkage (correlation coefficient and modelling), additional certainty about the metabolic involvement of the markers with respect to TU treatment was obtained. (DOCX) [file pone.0195351.s005.docx]

Discovery of Urinary Biomarkers to Discriminate Between Exogenous and Semi-Endogenous Thiouracil in Cattle: A Parallel-Like Randomized Design

Thiouracil administration in cattle and urinary biomarkers

Lieven Van Meulebroek^a^, Jella Wauters^a^, Beata Pomian^a^, Julie Vanden Bussche^a^, Philippe Delahaut^b^, Eric Fichant^b^, Lynn Vanhaecke^a^

^a^ Ghent University, Faculty of Veterinary Medicine, Department of Veterinary Public Health and Food Safety, Laboratory of Chemical Analysis, Salisburylaan 133, 9820 Merelbeke, Belgium;

^b^ CER Groupe, Health Department, Rue Point du Jour 8, 6900 Marloie, Belgium.

**S4 Table. Filtering of candidate markers for cows.**

| **compound ID** | **sensitivity  (%) (n = 65)** | **specificity  (%) (n = 21)** | **correlation coefficient (τ)** | **OPLS-based selection** |
| --- | --- | --- | --- | --- |
| 160 | 100 | 100 | 0.638 | ✓ |
| 405 | 100 | 100 | 0.938 | ✓ |
| 406 | **76.9** | 100 | 0.941 | ✓ |
| 445 | 81.5 | 95.2 | 0.921 | ✓ |
| 457 | 100 | 95.2 | 0.934 | ✓ |
| 875 | **69.2** | 100 | 0.501 | ✓ |
| 920 | 98.5 | 100 | 0.635 | ✓ |
| 1873 | 100 | 100 | 0.674 | ✓ |
| 6001 | 95.4 | 100 | 0.522 | ✓ |
| 6130 | 95.4 | 100 | 0.566 | ✓ |
| 7106 | 90.8 | 100 | **0.371** | ✓ |
| 7334 | 93.8 | 100 | 0.632 | ✓ |
| 7552 | 93.8 | 100 | 0.584 | ✓ |
| 7676 | 98.5 | 100 | 0.586 | ✓ |
| 11934 | 30.8 | 100 | **0.253** | **✗** |
| 466 | 96.9 | 95.2 | 0.985 | ✓ |
| 467 | 100 | 95.2 | 0.982 | ✓ |
| 468 | 100 | 100 | 0.986 | ✓ |
| 2385 | 100 | 100 | 0.681 | ✓ |
| 2386 | 89.2 | 100 | 0.667 | ✓ |
| 4357 | 100 | 100 | 0.699 | ✓ |
| 4359 | 98.5 | 100 | 0.612 | ✓ |
| 4361 | 100 | 100 | 0.690 | ✓ |
| 4466 | 100 | 100 | 0.589 | ✓ |
| 5119 | 100 | 100 | 0.631 | ✓ |
| 5120 | 93.8 | 100 | 0.624 | ✓ |
| 6145 | 92.3 | 100 | **0.313** | **✗** |
| 6152 | 90.8 | 95.2 | **0.135** | **✗** |
| 9176 | **69.2** | 95.2 | **0.299** | **✗** |
| 9177 | 96.9 | 100 | **0.341** | **✗** |
| 9179 | 96.9 | 95.2 | 0.651 | ✓ |
| 16756 | 98.5 | 95.2 | 0.538 | **✗** |
| 20949 | 100 | 100 | 0.758 | ✓ |
| 20952 | 100 | 95.2 | 0.741 | ✓ |
| 20954 | 100 | 95.2 | 0.702 | ✓ |
| 21117 | 96.9 | 95.2 | **0.389** | **✗** |
| 21221 | 100 | 100 | **0.232** | **✗** |
| 22297 | 96.9 | 100 | 0.564 | **✗** |
| 22303 | 92.9 | 100 | **0.343** | **✗** |
| 22481 | 100 | 100 | 0.613 | **✗** |
| 22670 | 96.9 | 100 | 0.610 | ✓ |
| 22933 | 100 | 100 | 0.506 | **✗** |
| 23815 | 89.2 | 100 | **0.387** | **✗** |
| 23951 | 96.9 | 100 | **0.319** | **✗** |
| 24312 | 96.9 | 100 | **0.388** | **✗** |
| 26256 | 93.8 | 95.2 | **0.340** | **✗** |
| 26257 | 98.5 | 95.2 | **0.337** | **✗** |
| 27655 | 96.9 | 95.2 | **0.337** | **✗** |
| 28490 | 90.8 | 100 | **0.152** | **✗** |
| 29853 | 93.8 | 100 | **0.388** | **✗** |
| 30488 | 96.9 | 100 | **0.213** | **✗** |
| 32967 | 93.8 | 100 | **0.325** | **✗** |
| 33682 | 100 | 100 | **0.354** | **✗** |

Selection of candidate markers based on the sensitivity and specificity as determined for the TU treated cows and those that received the rapeseed-enriched diet. In addition, based on metabolic linkage (correlation coefficient and OPLS modelling), additional certainty about the metabolic involvement of the markers with respect to TU treatment was obtained.
