# Supplementary material for: Discovery of urinary biomarkers to discriminate between exogenous and semi-endogenous thiouracil in cattle: A parallel-like randomized design
Source: PLoS One. 2018 Apr 12;13(4):e0195351. doi: 10.1371/journal.pone.0195351 (PMC5896977; doi:10.1371/journal.pone.0195351)

Discovery of Urinary Biomarkers to Discriminate Between Exogenous and Semi-Endogenous Thiouracil in Cattle: A Parallel-Like Randomized Design

Thiouracil administration in cattle and urinary biomarkers

Lieven Van Meulebroek^a^, Jella Wauters^a^, Beata Pomian^a^, Julie Vanden Bussche^a^, Philippe Delahaut^b^, Eric Fichant^b^, Lynn Vanhaecke^a^

^a^ Ghent University, Faculty of Veterinary Medicine, Department of Veterinary Public Health and Food Safety, Laboratory of Chemical Analysis, Salisburylaan 133, 9820 Merelbeke, Belgium;

^b^ CER Groupe, Health Department, Rue Point du Jour 8, 6900 Marloie, Belgium.

**S1 Fig.** **Chromatograms for the eleven metabolite markers.** Chromatograms were acquired from samples from TU treated animals, with characteristic information about the retention time (RT), *m/z*-value, ionization adduct, intensity, and tentative chemical configuration.


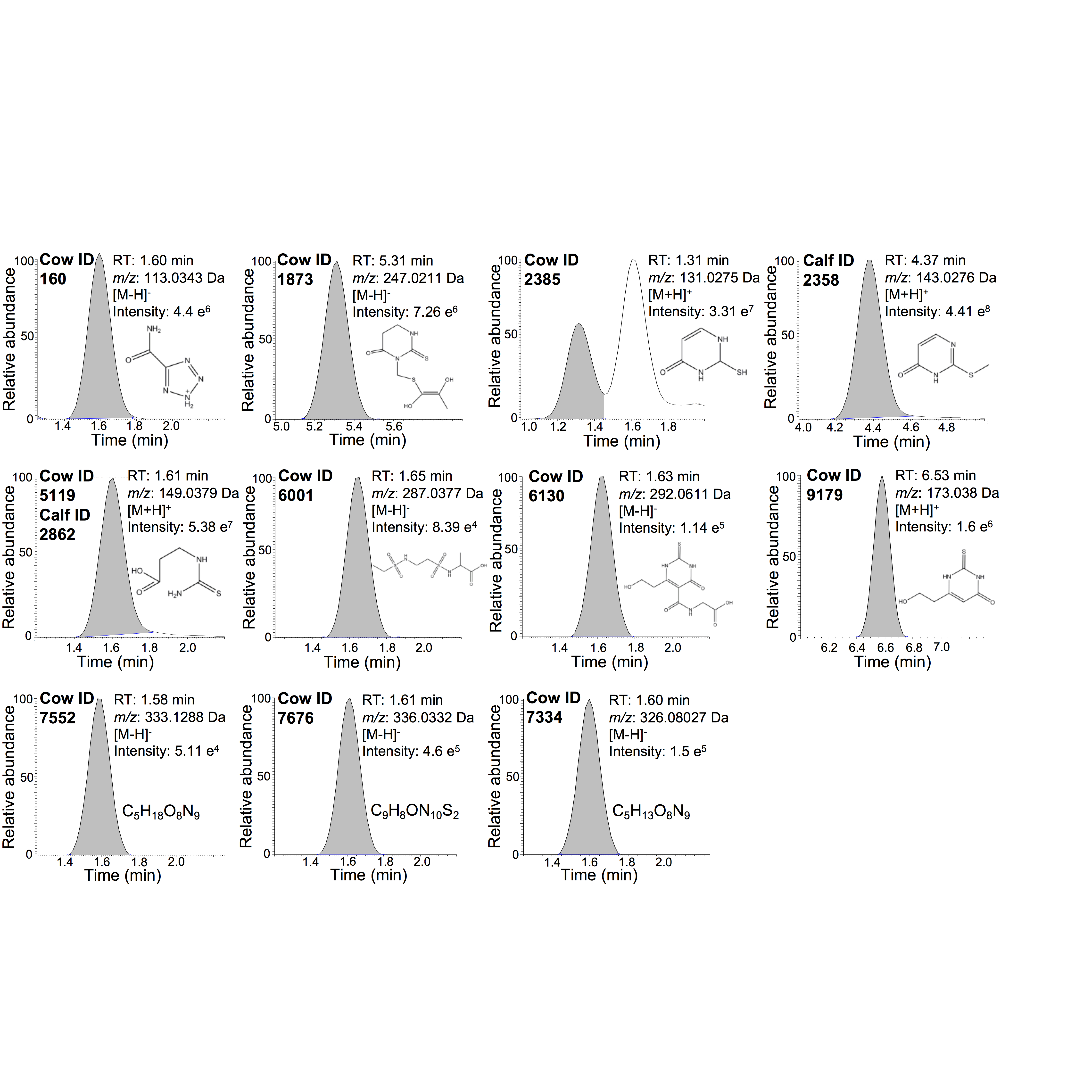

Supplement: S1 Fig — Chromatograms were acquired from samples from TU treated animals, with characteristic information about the retention time (RT), m/z-value, ionization adduct, intensity, and tentative chemical configuration. (DOCX) [file pone.0195351.s006.docx]
